# Behavioral and Reproductive Response of White Pine Weevil (*Pissodes strobi*) to Resistant and Susceptible Sitka Spruce (*Picea sitchensis*)

**DOI:** 10.3390/insects1010003

**Published:** 2010-08-19

**Authors:** Jeanne A. Robert, Jörg Bohlmann

**Affiliations:** Michael Smith Laboratories, University of British Columbia, Vancouver, BC V6T 1Z4, Canada; E-Mail: jrobert@unbc.ca

**Keywords:** *Pissodes strobi*, white pine weevil, host selection, insect ovary development, *Picea sitchensis*, Sitka spruce, conifer defense

## Abstract

White pine weevil (*Pissodes strobi,* Peck.) is a native forest insect pest in the Pacific Northwest of North America that attacks species of spruce (*Picea spp.*) and pine (*Pinus spp.*). Young Sitka spruce [*Picea sitchensis* (Bong.) Carr.] trees are particularly susceptible to weevil attack. Pockets of naturally occurring Sitka spruce resistance have been identified in high weevil hazard areas in coastal British Columbia. In this study, we characterize behavioral, physiological and reproductive responses of weevils to an extremely resistant Sitka spruce genotype (H898) in comparison to a highly susceptible genotype (Q903). The experiments relied on a large number of three-year-old clonally propagated trees and were therefore restricted to two contrasting Sitka spruce genotypes. When exposed to resistant trees, both male and female weevils were deterred during host selection and mating, females showed delayed or reduced ovary development, and successful reproduction of weevils was prevented on resistant trees.

## 1. Introduction

White pine weevil (*Pissodes strobi,* Peck.) attacks several ecologically and economically important conifer hosts [1]. Young Sitka spruce [*Picea sitchensis* (Bong.) Carr.] trees are particularly susceptible to feeding adult insects and their larvae [2]. Adult weevils emerge in the spring from the duff of the forest floor to search for the apical stem sections of young host trees on which to feed and mate. In the late spring and early summer, female weevils oviposit into the phloem tissue (outer stem tissues underneath the cortex) of the fully developed uppermost stem section (apical shoot leader) of the host tree. The developing larvae feed on the nutrient rich phloem as well as on the adjacent cambial zone (a thin layer of meristematic cells responsible for stem growth) and outer layers of wood forming tissue. By destroying these tissues that are essential for apical growth of the young tree as well as for water and nutrient transport to the growing shoot tip, weevil larvae can kill the infested apical stem section and prevent new growth from the shoot tip. Stem deformation occurs when one or multiple lateral branches below the attacked stem section assume apical dominance. Weevil attack causes growth losses and potentially tree and stand mortality [3]. Because of its extreme susceptibility to attack by white pine weevil, Sitka spruce is no longer commercially planted in substantial numbers in its natural range of distribution in the Pacific Northwest of North America. Introduction of white pine weevil to other parts of the world, e.g., by transport of infested plant or soil material, could cause disaster to other highly susceptible host species such a Norway spruce [4] which dominates many of the conifer forests of Europe and Asia.

Sitka spruce trees showing natural resistance to weevils have been identified in coastal British Columbia (BC, Canada), and resistant genotypes have been successfully confirmed in replicated clonal field trials as part of the Sitka spruce breeding program of the BC Ministry of Forests and Range (BCMFR) [5]. From these trials, the genotype H898 stood out as being almost completely resistant to weevil attack [2,3,4,5]. Characteristics that may contribute to resistance of Sitka spruce to weevil attack include a number of physical and chemical defenses, and host resistance may affect weevil behavior and reproductive fitness [4,5,6]. The H898 genotype originates from the Haney area of the lower mainland of coastal BC, an area of high weevil hazard. In contrast to the H898 genotype of Sitka spruce, which displays rare resistance to weevils, the genotype Q903 represents the characteristic high susceptibility to weevils. The Q903 genotype originates from Haida Gwaii (formerly called Queen Charlotte Islands) off the coast of B.C., which is free of any known weevil pressure.

The Sitka spruce genotypes H898 and Q903 have been propagated in the form of young grafted sapling trees (3 years of growth since the grafting of one-year-old shoots) and provided a unique resource for experiments with weevils that required a large number of clonally propagated trees. In a pair-wise comparative study using the highly resistant H898 genotype and the highly susceptible Q903 genotype, we characterized the behavioral and reproductive response of weevils, including feeding patterns, host choice, ovary development, egg laying behavior, and larval development. The results of this study showed that weevils respond to the highly resistant Sitka spruce genotype H898, but not the susceptible Q903 genotype, with deterrence of both male and female weevils during host selection and mating, delayed ovary development in females, and disruption of egg development, hatching, or development of larvae.

## 2. Materials and Methods

### 2.1. Plant Materials and Maintenance

The BCMFR (Cowichan Lake Research Station, Vancouver Island, BC) supplied three-year-old grafted Sitka spruce clones of the highly resistant (H898) genotype originating from the Haney area east of Vancouver (49°14’N: 122°36’W) and the highly susceptible (Q903) genotype originating from an area with no history of weevil attack, Haida Gwaii (formerly called Queen Charlotte Islands) on BC’s west coast (53°55’N: 132°05’W) [2]. Trees were watered regularly and carefully maintained in 4 liter pots outside on the University of British Columbia (UBC) campus. One month prior to experiments, healthy trees with good leader growth were moved into the ambient temperature and light zone of the UBC greenhouse [7]. Leader diameter was measured for each tree at the beginning of each experiment. In order to best simulate the weevil’s normal host selection, mating and oviposition conditions, the no-choice experiments were conducted in April–May 2007, the choice experiments (with mixed females and males) were conducted in May 2008, and the male only choice experiment was conducted in May 2009.

### 2.2. Insect Rearing and Maintenance

Weevils were collected by harvesting apical shoot leaders of infested Sitka spruce trees before insect emergence in the fall. Leaders were collected from two BCMFR research plantations in the Campbell River area on Vancouver Island (49°57’N, 125°16’W). Adult weevils were left to emerge in 16 L ventilated plastic buckets placed in a controlled environment chamber (Conviron chamber 8TC10, 2006) set to 22 °C with an 18h:6h light-dark cycle. Every second day, emergent adult weevils were removed from the buckets and transferred to fresh Sitka spruce cuttings of uncharacterized mixed origin. The weevils remained for 5 weeks in the emergence chamber before being transferred to a controlled environment chamber (Conviron) set for a 6 °C day and a 4 °C night regime. Fresh spruce clippings were added as food source at 10–14 day intervals. For all experiments, weevils were separated into males and females according to Harman and Kulman [8] and Lavallée *et al.* [9].

### 2.3. Assessment of Constitutive and Weevil-Induced Tree Defense Response

We characterized constitutive and weevil-induced defenses in the resistant and susceptible genotypes when exposed to weevil feeding and oviposition by assessing the constitutive resin canals (all resin canals present in the bark tissue), the development of traumatic resin ducts (resin canals produced in the cambium shortly after weevil attack and ultimately embedded in the xylem) in response to weevil feeding, and by characterizing the constitutive and weevil-induced monoterpene profiles for each genotype versus untreated control trees.

### 2.4. Assessment of Constitutive and Traumatic Resin Ducts

Following earlier work on Sitka spruce terpenoid defenses [4,7,10], we measured the number and area of constitutive cortical resin ducts in the phloem tissue (outer stem tissue) and the number and area of weevil-induced, newly formed traumatic resin ducts in the xylem (inner stem tissues) in the leader. Two adult female weevils and two adult male weevils were caged on the apical leader and highest interwhorl section of each tree. We sampled three weevil-attacked trees and three control (unattacked) trees per genotype after 22 days of continuous weevil exposure. A small (1 cm) section from the base of the apical leader was cut into 1mm thick slices and embedded in resin in three steps over four days: fixation, dehydration and infiltration of resin. Each tissue sample was fixed immediately after harvesting in 1 mL 4% formaldehyde in 50 mM PIPES (sodium salt, minimum 99% titration, SIGMA) buffer (pH 7.2) and incubated overnight in a vacuum at room temperature. The next day, fixation was completed by washing twice with 1 mL PIPES buffer for 10 minutes each wash. The tissue was then dehydrated in 1 mL absolute ethanol in nine thirty minute wash intervals beginning with a 10% ethanol in 50 mM PIPES buffer (pH 7.2) and working up to 90% ethanol solution. The dehydration step was then completed with two hour-long washes with 1 mL absolute ethanol, and then the samples were left in 1 mL absolute ethanol overnight. The next day, resin infiltration was conducted in thirty minute wash intervals beginning with 1 drop of LR White resin (hard grade acrylic resin, London Resin Company Ltd.) per 1 mL absolute ethanol up to 5 drops resin per milliliter of ethanol. Then, six one-hour washes were conducted beginning with 15% resin (by volume) in ethanol up to 60% resin in ethanol. Samples were left in 60% resin solution over night. The infiltration was continued the next day in three, one-hour washes beginning with 70% resin in ethanol up to 90% resin in ethanol. Two two-hour washes with 100% resin completed the infiltration. The samples were left one final night in 100% resin. The next day, the samples were washed in 100% resin before pouring into molds and baking for overnight at 60 °C to solidify the resin in preparation for sectioning. Each sample was cut into 600 nm thick sections which were viewed using a Zeiss Axioskop 2 MOT compound microscope and photographed using a Zeiss AxioCam HRc camera outfitted with a TV2/3" C > 0.63x lens. Two photos, one at 5X magnification and the other at 20X magnification (to ensure the correct identification of newly formed traumatic resin ducts), were taken of each section. The images were analysed using AxioVS40 V 4.6.1.0 (Carl Zeiss Imaging Solutions, 2002–2007). The inner bark thickness (phloem plus cortex, excluding periderm) was measured on one randomly chosen slice by averaging the thickest and the thinnest part of the inner bark on each section. The number of traumatic resin ducts produced in response to the weevil attack was determined from the photos of each cross section sampled after 22 days of weevil feeding. In order to adjust for the varying size of the cross sections, traumatic resin duct abundance was expressed as number of ducts per millimeter cambium circumference.

### 2.5. Analysis of Monoterpene Profiles

Extraction and analysis of monoterpene compounds present in the leader tissue was conducted for 3 replicates (destructively sampled at each timepoint) of each Sitka spruce genotype at 2, 5, 10, 15 and 22 days of continuous weevil exposure. Two adult female weevils and two adult male weevils were caged on the leader and first interwhorl (previous year’s leader growth) of an individual tree. Both the presence and amount of monoterpene (μg/g dry weight tissue) was analysed using gas chromatography (GC) (Agilent 6890A series). The extraction method used is based on Lewinsohn *et al.* [11] and conducted as described in Martin *et al.* [12] with the following modifications. As in the original protocol, approximately 1 cm length of leader tissue (~0.2 grams dry weight) was cut lengthwise and immersed in 1.5 mL *tert*-butyl methyl ether (Chromasolv Plus, for HPLC, 99.9% MTBE, Sigma-Aldrich), containing 100 µg/mL isobutyl benzene (Fluka) as an internal standard, and shaken overnight at room temperature. The next day, the extract was washed with 0.3 mL of 0.1 M (NH_4_)_2_CO_3_ (pH 8.0). The extracted tree tissue was removed, dried at room temperature in the fumehood for one week, and then weighed.

Identification of monoterpene compounds was achieved through the comparison of compound retention time with the retention time of commercially available authentic standards. Identities of the compounds were confirmed using GC coupled with mass spectrometry (MS) (5973N mass selective detector, quadropole analyzer, electron ionization, 70 eV). The following program was used to separate monoterpenes on a SGE Solgel-Wax capillary column (Mandel Scientific SG-054796, 250 µm diameter, 30 m length, and 0.25 µm film thickness): the 40 °C initial temperature was increased by 3 °C min^−^^1^ to 110 °C, then increased at 10 °C min^−^^1^ to 180 °C, then finally increased by 15 °C min^−^^1^ to 260 °C held for 15 minutes (total run time is 50.67 minutes). The initial injection temperature was set at 250 °C, and the initial flow rate was 1 mL He min^−^^1^. Stereochemistry of the compounds was determined where authentic standards were available on a Cylcodex-B capillary column (J&W 112-2532, 250 µm diameter, 30 m length, and 0.25 µm film thickness) using the following temperature program: the 55 °C initial temperature was increased by 1 °C min^−^^1^ to 100 °C, then increased at 10 °C min^−^^1^ to 230 °C held for 10 minutes (total run time is 69.00 minutes). The initial injection temperature was set at 230 °C, and the initial flow rate was 1mL He min^−^^1^. Response factors were calculated for each compound and the compounds were quantified using a known concentration of internal standard, isobutyl benzene. The amount of compound (µg) per gram dry weight of the tissue extracted was calculated and used as the final value for comparisons.

### 2.6. Assessment of Weevil Responses to Resistant and Susceptible Tree Genotypes 

We conducted a series of choice and no-choice experiments to assess the responses of weevils to resistant and susceptible tree genotypes. In the no-choice assays, weevils were caged on either individual resistant or susceptible trees. In the choice experiments weevils were free to move between the resistant and susceptible genotypes. We tracked individual weevil movement over time and assessed the number of feeding holes, ovary development, oviposition, and larval numbers surviving on resistant versus susceptible trees.

### 2.7. Assessment of Insect Ovary Development

Ovary development was recorded based on Pernal and Currie [13] who developed a method for assessment of ovary development for honey bees that was originally derived from Velthus [14]. Assessment of ovary development was based on information on white pine weevil ovary development [15] as well as *Sitophilus oryzae* (rice weevil) ovarian physiology was obtained from Perez-Medonza *et al.* [16] and Khan and Musgrave [17].

Ovary development was scored in dissected insects using a dissecting microscope (Wild M38, Heerbrugg Switzerland, 40× magnification) as one of three categories: low development, moderate development, or mature. Low development includes undeveloped ovaries (small transparent ovarioles) as well as the early initiation of oogenesis (cells beginning to swell at the germarian and follicles moving into the vitellarium); moderate development includes follicles that are still in the vitellarium, they are round or bean-shaped and follicular epithelium is still visible; and mature ovaries were identified by highly developed (mature) sausage-shaped eggs in the lower vitellarium, in the lateral oviduct or the common oviduct. The ovary development category was assigned based on the highest level of development noted during examination of the ovaries as the previous stages are usually visible if the later stages are present.

### 2.8. Assessment of Feeding and Oviposition in No-Choice Assays

This experiment was designed to characterize weevil feeding patterns and oviposition on the resistant versus susceptible trees when given no choice. For the experiment, weevils were removed from 4 °C, placed in petri dishes lined with moistened paper towel, and starved for 48 h prior to placing them on trees. Two adult female weevils and two adult male weevils were caged on the leader and first interwhorl of each tree. Three trees per genotype were analysed for the number of feeding and oviposition holes after 2, 5, 10, 15 and 22 days of continuous weevil exposure.

### 2.9. Assessment of Ovary Development in No-Choice Assays

The experimental design above was repeated but with an extended timecourse (sampling after 2, 7, 14, 21 and 28 days of continuous weevil exposure) and a larger number of weevils per tree (3 adult females and 2 adult males per tree) in order to better assess ovary development, and the number of eggs and surviving larvae for adults caged on the resistant versus the susceptible tree genotype. For the weevils recovered at each timepoint, weevil weight and ovary development were recorded. At the beginning of the experiment, 15 female weevils were dissected to ensure that no ovary development had occurred prior to the experiment after 48 hours starvation and before feeding on the experimental tree tissue.

### 2.10. Assessment of Feeding and Oviposition in Choice Assays

The objective of this experiment was to determine whether adult weevils will occupy, feed, and oviposit preferentially when given a choice between trees of the highly susceptible genotype and the highly resistant genotype. Six trees (3 resistant and 3 susceptible) were placed in a 1 m width × 1.5 m length × 1.5 m height mesh cage. The trees were placed in two rows with 3 trees in each row. The genotypes were altered systematically. Weevils were removed from 4 °C, placed in petri dishes lined with moistened paper towel, and starved for 48 hours prior to placing them on trees. The experiment was conducted in two variations, one with a mix of adult males and females, and one with adult male weevils only.

In the experiment with males and females mixed, 3 female weevils and 2 male weevils were placed on each tree in the mesh cage at the beginning of the experiment (18 female weevils and 12 male weevils in total). Individual female weevils were identified with a color code of two colored spots of oil-based paint (Testor Model Paint) applied to the elytra. The location of each female weevil was recorded at 2–3 day intervals for a total of 23 days (Day 2, 5, 7, 9, 12, 14, 16, 19, 21, 23). The number of males observed on each tree was also recorded at each timepoint. On final day (day 23) of the timecourse, the number of feeding and oviposition holes was counted on the leader and the first interwhorl below the leader. The leader and the interwhorl tissue was dissected in order to record the number of eggs and larvae beneath the bark.

In the experiment with males only, the experimental conditions were identical as described above except that 5 adult male weevils were placed on each tree at the beginning of the experiment. Each male was marked with one spot of yellow paint to increase visibility. Again, the number of males on each genotype was recorded at each timepoint (Day 2, 5, 7, 9, 12, 14, 16, 19, 21, 23) and the number of feeding holes was recorded for each genotype at the end of the experiment on Day 23.

### 2.11. Statistical Analyses

Tests were conducted using SYSTAT 11.0 (Systat Software Inc. 2004). Where reported, the analyses satisfied the assumptions for both ANOVA (normality, independence of cases, and equality of variance) and Pearson χ^2^-squared analysis (independence of cases) [18].

## 3. Results

### 3.1. Assessment of Host Defense Responses in Weevil-Resistant H898 and -Susceptible Q903 Sitka Spruce Genotypes

**Figure 1:**
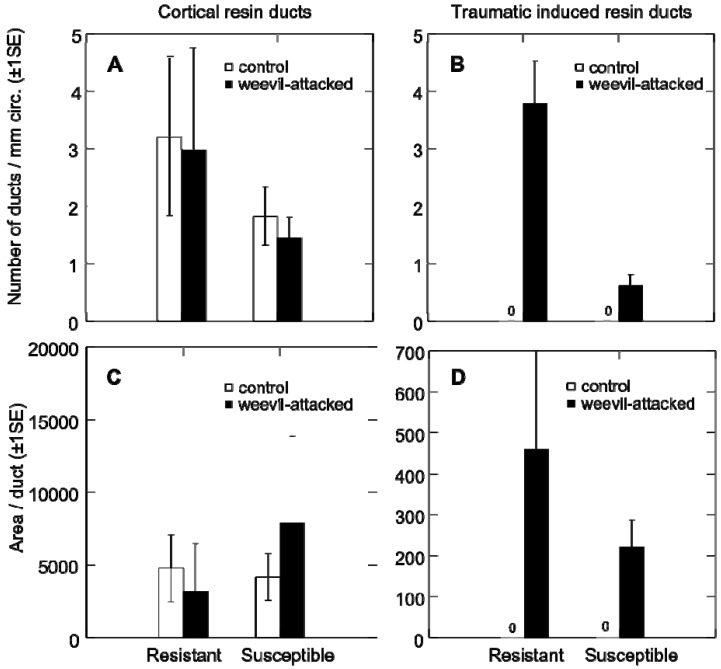
The number and area of cortical and induced resin ducts for weevil-attacked and control trees for resistant versus susceptible tree genotypes. (A) The average number of constitutive cortical resin ducts; and (B) induced traumatic resin ducts per millimeter stem circumference (± 1SE); (C) The average area per duct (± 1SE) of cortical resin ducts; and (D) induced traumatic resin ducts for resistant and susceptible Sitka spruce genotypes.

Prior to using the clonally propagated resistant and susceptible trees in experiments to study weevil behavior, physiology and reproductive success, we assessed defense related traits. Resistant and susceptible trees showed substantial differences in their defense response of weevil induced traumatic resin duct formation (Figure 1B and 1D). The resistant genotype showed a significantly larger number of traumatic resin ducts at day 22 of weevil feeding relative to untreated control trees (tree genotype: *F*_1, 7_ = 102.8, *p* < 0.001; treatment: *F*_1, 7_ = 197.1, *p* < 0.001), whereas the susceptible genotype showed almost no traumatic resin duct formation. The number and area of constitutive cortical resin ducts were not significantly different between the two genotypes (Figure 1A and 1C).

As bark thickness can play a role in weevil oviposition behavior, we measured both the diameter and average bark thickness of the apical shoot. The leader diameter of resistant trees (average ± 1SE: 4.44 mm ± 0.14) was consistently smaller than that of the susceptible trees (6.15 mm ± 0.18) (*F*_1, __69_ = 38.1, *p* < 0.001). However, there was no difference in bark thickness of the leaders of the two genotypes (*F*_1, 69_ = 1.3, *p* = 0.253) (resistant: 0.82 mm ± 0.03, susceptible 0.87 mm ± 0.03).

**Figure 2:**
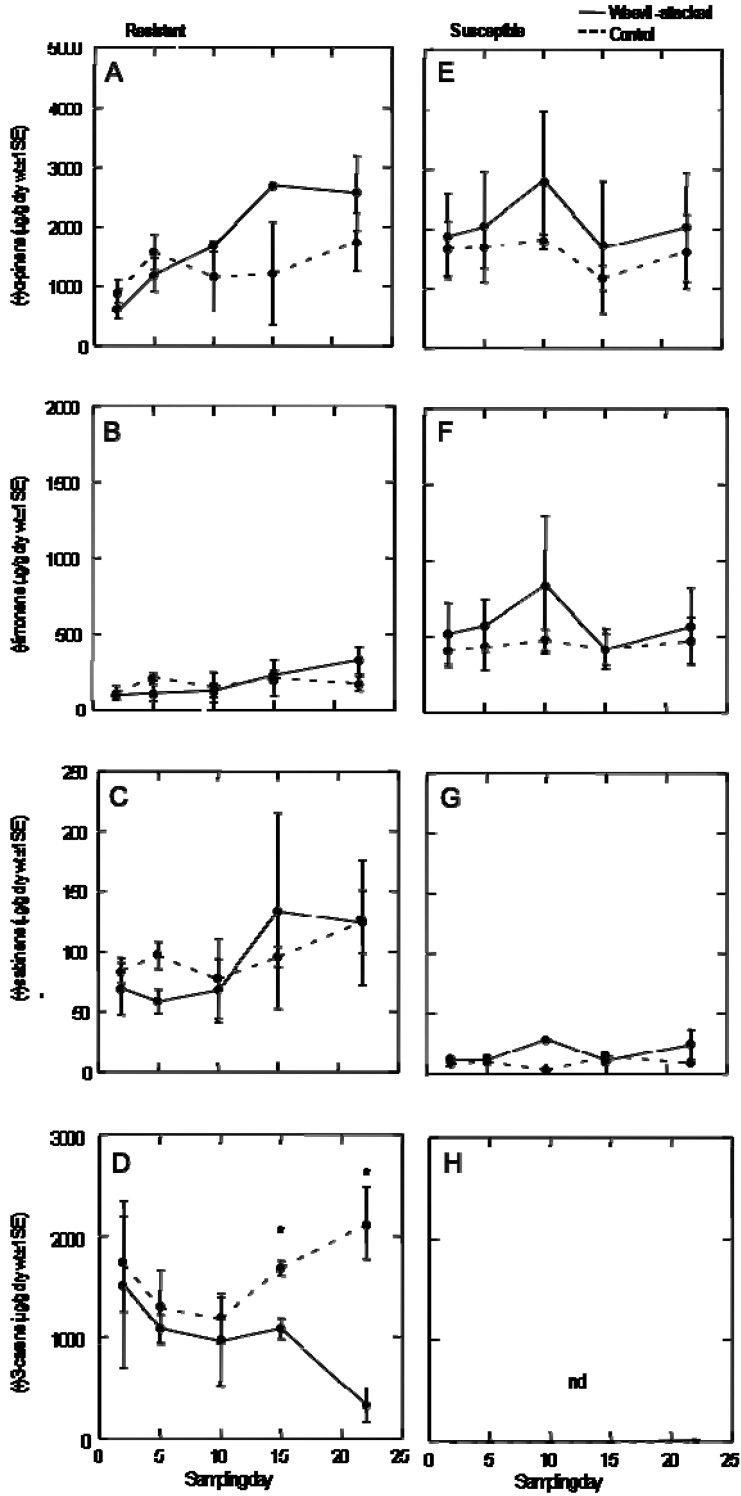
The resistant and susceptible monoterpene profiles in response to weevil feeding. The average amount of monoterpene measured (µg/g dry weight ± 1SE) on each sampling day for resistant (A-D) and susceptible (E-H) trees. Asterisk indicates a significant difference between treatment and control (p < 0.05). nd = not detected).

The monoterpene profiles over 22 days of continuous weevil feeding were variable but largely unchanged in either resistant or susceptible trees for the compounds we measured, specifically (+)-α-pinene, (−)-limonene, (+)-sabinene and (+)-3-carene (Figure 2). Most of the compounds did not change significantly with continuous weevil feeding in either genotype, but (+)-3-carene decreased significantly at day 15 (*F*_1, 3_ = 30.756, *p* < 0.012) and day 22 (*F*_1, 4_ = 35.524, *p* < 0.004) in the resistant genotype exposed to weevil feeding. Importantly, the monoterpene (+)-3-carene was not detected in Q903 consistent with recent observations of (+)-3-carene as an indicator for Sitka spruce resistance against white pine weevil [6].

### 3.2. Weevil Behavior and Ovary Development Affected by Resistant and Susceptible Host Trees in No-Choice Scenarios

**Figure 3:**
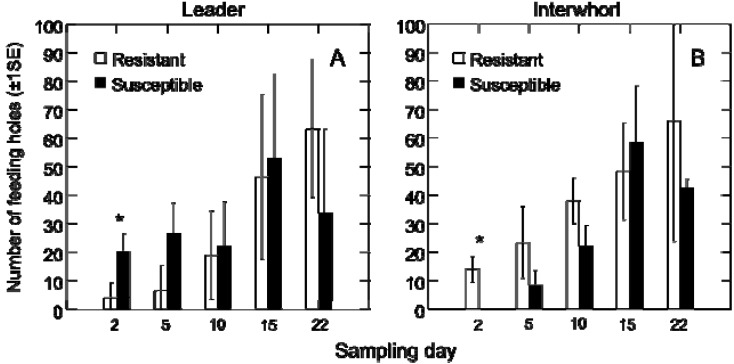
The number of feeding holes on the leader and interwhorl of the resistant and susceptible tree genotypes. (A) The number of feeding holes observed on resistant and susceptible trees (± 1SE) on the leader; and (B) on the interwhorl directly below the leader. An asterisk indicates a significant difference between genotypes (p < 0.05).

To test for differences in the feeding and oviposition behavior, or differences in ovary development, for weevils restricted either to the resistant genotype or the susceptible genotype. A differential feeding pattern between host genotypes was apparent, in particular, for the early timepoints of the experiment. On day two, marginally significantly fewer feeding punctures were found on the leader of the resistant trees than were counted on susceptible trees (*F*_1, 4_ = 7.0, *p* = 0.057) (Figure 3A). The opposite pattern was observed on the interwhorl directly below the leader (*F*_1, 4_ = 17.8, *p* = 0.013) (Figure 3B). These differences became indistinguishable by day ten as trees were destructively sampled at each point in the time course.

**Figure 4:**
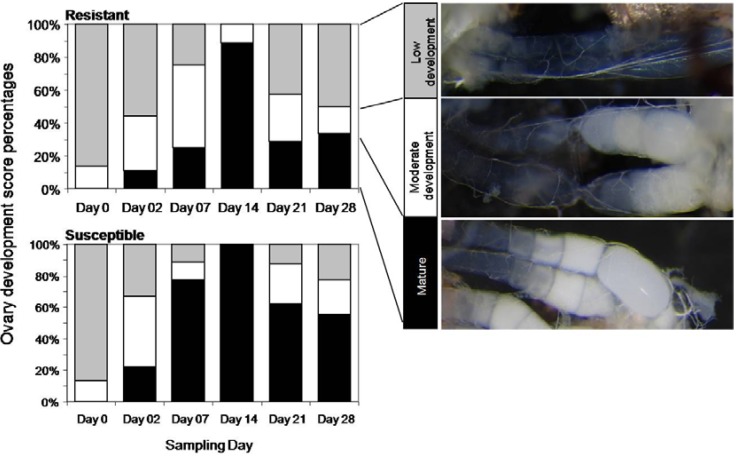
Weevil ovary development on resistant and susceptible tree genotypes. The percentage weevils with low (gray bars), moderate (white bars), or mature (black bars) ovary development for each sampling day. Weevils were restricted to either resistant host trees (top) or to susceptible host trees (bottom).

Ovary development was also different for weevils caged on either resistant or susceptible trees (Figure 4). Two days after exposure to host trees, 22% of the females on the susceptible genotype and 11% of the females on the resistant genotype contained mature eggs. By day seven, 78% of the females caged on susceptible trees contained mature eggs whereas only 25% of the females caged on resistant trees contained mature eggs. By day 14, 100% of the females caged on the susceptible trees contained mature eggs versus 89% on the resistant trees. After 21 days, 62% of the females caged on susceptible trees still contained mature eggs versus only 29% of females caged on the resistant trees.

### 3.3. Weevil Behavior and Reproductive Success Affected by Resistant and Susceptible Host Trees in Choice Scenarios

A set of choice experiments were performed in order to assess male and female host selection preferences and to assess oviposition choices and larval development on resistant versus susceptible trees. Overall, weevils were highly mobile. On average, a given female weevil was observed on 3.7 different trees. The average number of consecutive sampling days that a weevil was found on the same tree was 2.7 days. No weevils were found on the same tree throughout the complete period of 23 days.

**Figure 5:**
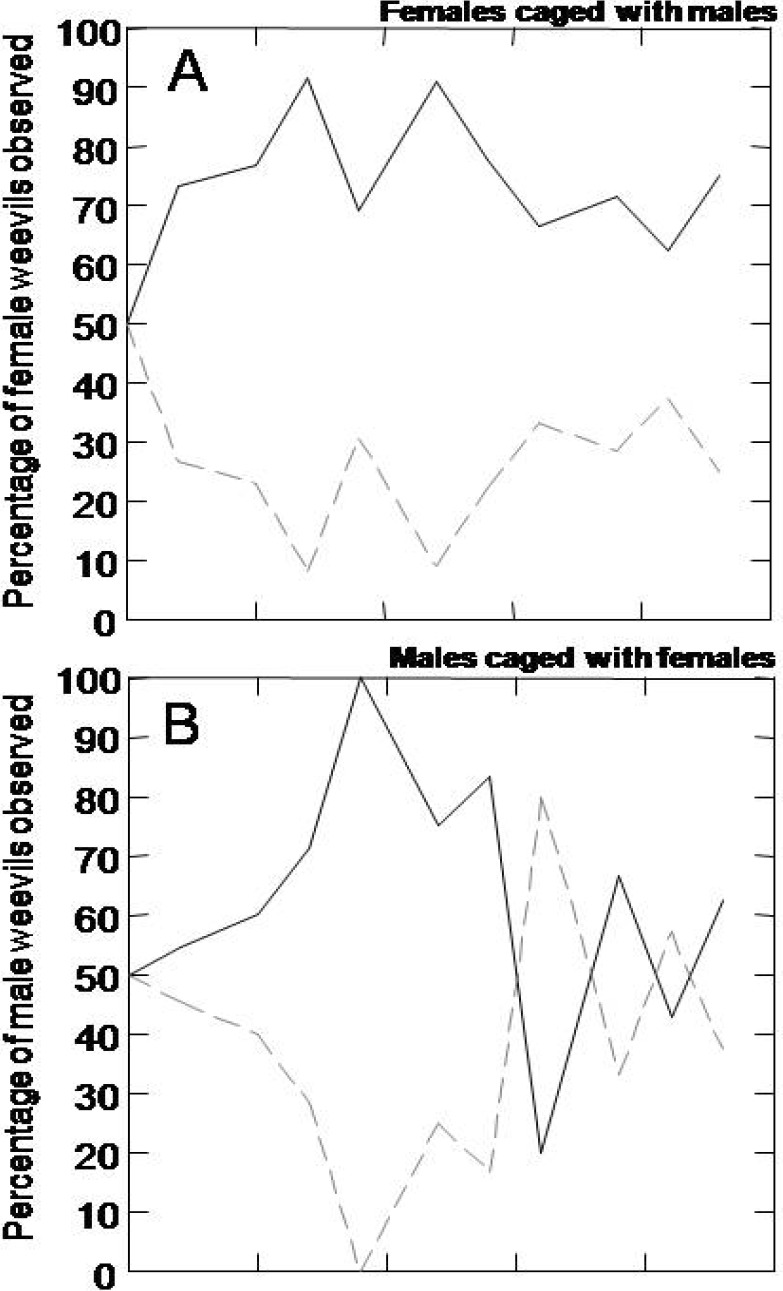

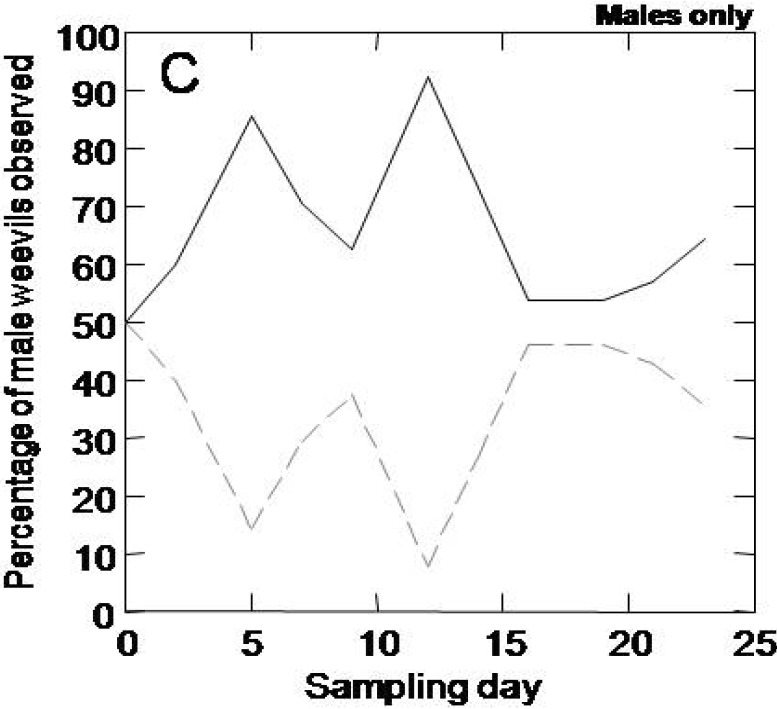
Female and male choice of resistant versus susceptible host tree genotypes. Weevil distribution on susceptible (solid lines) and resistant (dotted lines) trees in choice experiments. (A) The percentage of female weevils when mixed with male weevils; (B) male weevils when mixed with female weevils; and (C) male weevils only, observed on the susceptible and on the resistant genotypes at each sampling day.

Over the entire timecourse of the choice experiment, a higher number of female weevils were observed on the susceptible tree genotype (Figure 5A). Over 70% of the females had already moved to a susceptible tree by day two and this proportion remained fairly constant until the end of the experiment at day 23. Movement of male weevils reflected a pattern similar to that of the females for the first half of the time course until day 14 (Figure 5B). In contrast to the female weevils, after day 14 the males showed a random distribution on susceptible and resistant trees. We tested whether the presence of females was influencing male choice of susceptible versus resistant host trees. When males were caged without females (Figure 5C), the males again showed an initial preference for susceptible trees until day 15, then showed a more even distribution between resistant and susceptible trees.

**Figure 6:**
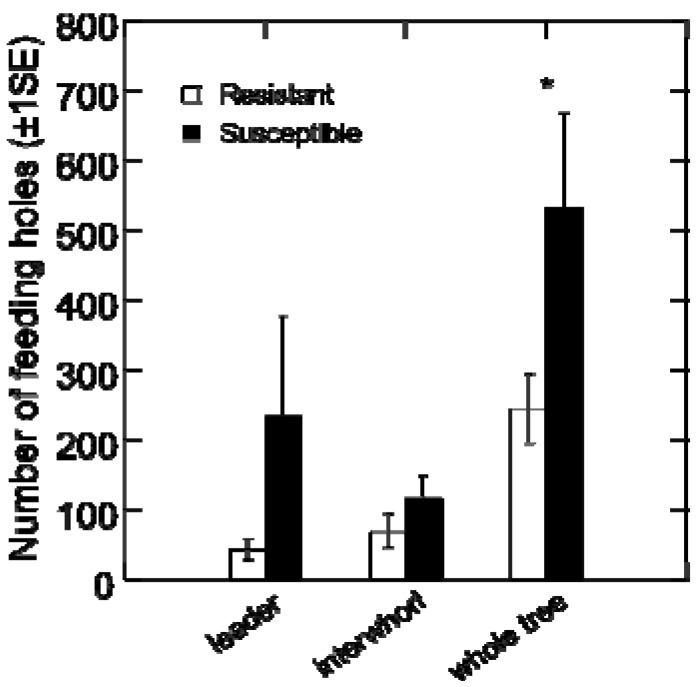
Feeding and oviposition punctures for weevils given a choice between resistant and susceptible host tree genotypes. The average number of feeding and oviposition holes (± 1SE) counted on the leader, interwhorl, and over the entire tree for the resistant and the susceptible genotypes over 23 days of continuous weevil exposure when the weevils were given a choice between tree genotypes.

Weevil feeding patterns and reproductive success were also different between resistant and susceptible host trees in the choice tests with female and male weevils combined. Generally, weevils fed less on the resistant trees as assessed by the number of feeding holes (*F*_1, 4_ = 7.084, *p* = 0.056 over the whole tree) (Figure 6). Successful reproduction, as assessed by development of larvae, occurred on the susceptible trees (average ± 1SE: 8.7 ± 0.9 larvae on the leader and 2.3 ± 1.4 larvae on the interwhorl), but no larvae were found on the resistant trees.

## 4. Discussion 

In a series of experiments we showed that weevils responded at multiple levels of behavior and reproductive activity differently to resistant and susceptible Sitka spruce sapling trees. Weevils were able to distinguish between the resistant Sitka spruce genotype H898 and susceptible genotype Q903 and when given a choice, showed preference for the susceptible host. When exposed to resistant trees, compared to exposure to susceptible trees, both male and female weevils were deterred during host selection and mating, females showed delayed or reduced ovary development, and no successful reproduction was found on the resistant genotype. We measured a number of parameters of the constitutive and weevil-induced defense response of the resistant and susceptible host genotype that might influence weevil behavior and physiology. Histological analysis showed that although leader diameter may have played a role in the weevil’s original choice not to feed on the resistant leader, bark thickness is not significantly different between the two genotypes at any of the timepoints and thus was likely not a factor in the observed feeding patterns. This is in contrast to the suggestion by Manville *et al.* [19] that female weevils are primarily using this characteristic to find and oviposit on leader tissue. Our data suggested that differential weevil feeding and oviposition between these resistant and susceptible genotypes were caused by variables other than bark thickness. Similar to previous research [20], histological analysis also showed that, in the resistant trees, the constitutive cortical resin duct defense system was bolstered by a strong response of induced traumatic resin duct formation. Traumatic resin canals in resistant genotypes were fully formed 22 days after weevil feeding whereas few induced traumatic resin canals were produced in the susceptible genotype. At the chemical level, the monoterpene profiles generally did not change significantly with continuous weevil feeding over 22 days except that (+)-3-carene, a compound associated with resistance in the area of origin of the H898 genotype [6], was significantly lower after extended periods of weevil feeding on resistant trees. It is possible that this compound, which was produced constitutively at relatively low levels, volatilizes from feeding holes at a rate that exceeds *de novo* biosynthesis. In contrast, more abundant compounds may be relatively less affected by loss from feeding holes or the loss may be compensated for by differential *de novo* formation [7,21].

Weevils avoided feeding initially on the apical shoot of the resistant genotype. This behavior could be a result of avoiding leader-specific feeding deterrents or toxins that were not present (or are present to a lesser extent) in the lower stem sections of resistant, and in leaders of susceptible genotype, respectively [22]. The amount or composition of constitutive resin in the leader of the resistant genotype may have initially deterred feeding. In addition, sclerids or stone cells have also been implicated in weevil resistance [23]; these may have been present in higher concentrations in the leader tissue of the resistant genotype. Another possible explanation for the differential feeding patterns is the presence of attractants or gustatory stimulants in susceptible trees. Trudel *et al.* [24] suggested chemical feeding stimulants in bark are necessary for growth and development of weevils reared in the laboratory.

When given a choice between the resistant and susceptible genotypes, female weevils were mobile and showed a clear preference for the susceptible genotype. This result suggests that female weevils may test a number of different potential hosts and may oviposit on more than one. In addition, male weevils also distinguished, but more transiently, between resistant and susceptible genotypes, both in the presence or absence of females. The majority of females remained on susceptible trees for the entire period of our experiments (23 days) whereas males showed a preference for susceptible trees only until day 15. Since males showed similar behavior with or without females, it is unlikely that males were simply following female cues when choosing susceptible trees. It is possible that females were more responsive to induced defense in the resistant trees than the males. Induced defense responses are well developed as early as one week after real or simulated insect attack (methyl jasmonate treatment) in Sitka spruce and Norway spruce [7,12,21], and female weevils can be more sensitive to volatile and gustatory cues than males [25,26].

In spite of a strong induced defense response in resistant trees, our results show that weevils were able to feed, develop ovaries and lay eggs on resistant trees, but ovary development was delayed and reduced, and no surviving offspring (larvae) developed on the resistant trees. Sahota *et al.* [27] also suggested that weevils feeding on resistant trees experience ovary regression or reduced ovary development. Our results suggest that although ovary development may be delayed for weevils feeding on this highly resistant genotype, it is not blocked. Sahota *et al.* [27] propose that reduced attack on resistant trees is an indirect result of the effects of resistance on female weevil physiology. However, it is unlikely that an effect on the physiology of ovary development or ovary regression is the only defense mechanism in the resistant H898 Sitka spruce genotype, since male weevils were also able to distinguish between resistant and susceptible trees in the presence and absence of females; and females were highly mobile, but showed low initial feeding on the resistant leader tissue in the no-choice experiment. We also showed that feeding punctures did not correlate with the number of surviving larvae [28]. Thus, interference with egg viability and larval development was likely to be another major factor in the resistance of the resistant H898 genotype.

Even though silvicultural treatments such as shading and planting of non-host species can reduce productivity losses associated with weevils, these interventions are not sufficient to enable commercial-scale regeneration of Sitka spruce. Two areas on the southern mainland, Haney (the origin of the H898 genotype) and Squamish, were noted in particular to have fewer than average attacks per tree even though these areas are rated as high weevil hazard areas [29]. Although the H898 genotype represents an extreme resistance in a generally highly susceptible host species, the identification and targeted selection of traits that are responsible for the rare and extreme resistance of genotypes like H898 are an important goal in tree breeding programs. Tree breeding for resistance using genotypes like H898 as a foundation, could ultimately enable the re-introduction of conifers like Sitka spruce, a commercially valuable, and now largely absent, native tree species into coastal British Columbia.

## 5. Conclusions

In summary, female and male weevils show differential responses to resistant (H898) and susceptible trees (Q903) at multiple levels of host selection, feeding activity, and reproduction. Along with further identification of host resistance traits (e.g., [4,5,6]), in future work, it may be possible to associate individual host resistance traits with individual behavioral, physiological or reproductive responses of male and female weevils.
